# Pretend play in children with a congenital visual impairment

**DOI:** 10.3389/fpsyg.2025.1535086

**Published:** 2025-04-30

**Authors:** Stefano Federici, Alessandra Bardin, Camilla Borsini, Elisa Delvecchio, Alessandro Lepri, Federica Morelli, Ilaria Scognamillo, Elena Cocchi, Luca Santini, Sabrina Signorini

**Affiliations:** ^1^Department of Philosophy Social and Human Sciences and Education, University of Perugia, Perugia, Italy; ^2^Developmental Neuro-Ophthalmology Unit, IRCCS Mondino Foundation, Pavia, Italy; ^3^Service des Troubles du Spectre de l'Autisme et Apparentés, Département de Psychiatrie, Lausanne University Hospital (CHUV), Lausanne, Switzerland; ^4^Department of Brain and Behavioural Sciences, University of Pavia, Pavia, Italy; ^5^Istituto David Chiossone Onlus, Genoa, Italy

**Keywords:** visual impairment, emotion regulation, pretend play, creativity, rehabilitation

## Abstract

**Background:**

Modern theories embrace a conception of pretend play as a behavior closely related to exploration, curiosity, and the affective domain, as well as problem solving and creative thinking. Although a widely studied construct, pretend play in children with a visual impairment has received little research attention.

**Objective:**

This study examined the influence of congenital visual impairments on symbolic skills by comparing differences in pretend play between 31 children (aged 3–9 years) with moderate to severe visual impairment or blindness with typically developing peers.

**Methods:**

The Affect in Play Scale was used as a measure of pretend play. A storytelling task, a parent-reported questionnaire, and the Emotion Regulation Checklist were used to examine the relationships between pretend play, creativity, and emotion regulation in both groups.

**Results:**

Results indicated that typically developing children demonstrated higher pretend play skills than their blind and visually impaired peers (*p* < 0.001), but there was no correlation between severity of impairment and play skills. Storytelling skills also appeared to be impaired in the population of children with blindness/visual impairment (*p* < 0.05), suggesting a link between pretend play and creativity. The data also showed a trend of negative correlation between emotion dysregulation and pretend play and storytelling performance in the visually impaired group, emphasizing that greater dysregulation was associated with lower pretend play skills.

**Conclusion:**

Our study has highlighted the importance of focusing a rehabilitation pathway on improving pretend play skills in the context of visual impairment to promote the development of the individual, supporting both cognitive and emotional dimensions.

## 1 Introduction

Pretend play, or symbolic play, can be defined as the human ability to play with an object by imagining (symbolizing) that it (the symbol) is something else (Fein, 1987). The acquisition and maturation of symbolic skills appear to be central to the child’s developmental period from 2 to 9 years, characterized by the transition from “preoperational” to “operational” at about 6–7 years, according to Piaget (1964). Building on Piaget’s work, research has helped to highlight the close relationship between play and symbolization activities, and between play skills and cognitive skills. Through pretend play, the child creates a twin world that is connected to the real world and enriched with meaning. In effect, pretend play serves as a link between the external and internal worlds, allowing the child to experience and understand both realities (Archinto, 2001).

More modern theories embrace a conception of play, including pretend play, as a behavior closely related to exploration, curiosity, and the affective sphere, as well as problem solving, creative thinking, socialization, and the internalization of social roles (Stagnitti, 2004). The affective and emotional processes involved have been studied in greater depth since the 1990s, most notably by psychologist and psychotherapist Sandra Russ (1993, 2014). The author defined cognitive processes in play as organization, divergent thinking, symbolism, and imagination/fiction, while affective processes include expression of emotion, expression of affective themes, enjoyment, emotion regulation, and cognitive integration of affect (Russ, 2014).

Skills central to play activities also characterize the creative dimension, particularly the ability to tell a story, which is the verbal creation of a story using imaginative and emotional elements (Hoffmann and Russ, 2016). Hoffmann and Russ (2012) investigated the relationship between storytelling and pretend play, highlighting a strong correlation between these constructs. The authors found that children who were more imaginative in pretend play also emerged as more creative storytellers. In addition, studies (Berk et al., 2006; Golomb and Galasso, 1995; Hoffmann and Russ, 2012; Moore and Russ, 2008; Russ and Wallace, 2013) have shown that children who are better at expressing and modulating their emotions also perform better in play and storytelling. As also advocated by Anna Freud (1965), children can access their emotional experiences through pretend play, increasing their capacity for understanding and regulating emotions. Emotion regulation can be defined as the ability to manage one’s emotional experiences appropriately (Shields and Cicchetti, 1998). This ability is trained and fostered within the context of pretend play and is fundamental in promoting general well-being and adaptive functioning. Although emotion regulation has been related to a wide range of domains of functioning, including social, psychological, and physical well-being and academic performance, however, to our knowledge, only one study conducted by Chennaz et al. (2022) investigated the effects of visual experience and age in emotion regulation by comparing groups of children with different visual status and age. The study’s findings indicated that both blind and visually impaired children between the ages of 3 and 12 years exhibited significantly lower scores in emotion regulation ability compared to their sighted counterparts. However, the study noted that the impact of age on emotion regulation may vary among blind and visually impaired children. Specifically, the scores of blind children decrease with age, while those of visually impaired children become comparable to those of sighted children as they get older.

According to the World Health Organization [WHO]‘s International Classification of Diseases, 11th edition (ICD-11; World Health Organization, 2019/2021), vision impairments result when a condition affects the visual system and one or more of its functions. According to the ICD-11, visual impairment is measured with the best corrected visual acuity at a distance of 3 m and can be categorized as mild, moderate, severe, or blindness. A congenital visual impairment can have a considerable impact on individual development (Dale et al., 2017; Fazzi et al., 2002; Warren, 1994; World Health Organization, 2019), thus also on the maturation of complex and adequate play behaviors. It is well known that approximately 80% of sensory information is conveyed through the visual system, making it the fastest and most precise acquisition channel for humans (Ferrell and Spungin, 2011). Vision provides direct access to the environment (Lewis et al., 2000), facilitating the formation of mental representations and offering greater opportunities for incidental learning and deferred imitation (Ferrell and Spungin, 2011). The combination of these processes enables children to develop a pretend play space, characterized by the substitution of objects and the enactment of previously observed situations (Pellegrini, 2009).

Since play presents a progressive and staged pattern, it is possible to find a concordance between a specific developmental stage and the complexity of the play shown (Mazzeschi et al., 2016). First, an “exploratory-functional” type of play is found, typical of the “sensorimotor stage” (Piaget, 1964), which involves children in the 0–2 age group. In this stage, children’s attention is focused on objects, their own body or parts of it. They also perform manipulative and observational actions that reveal the sensory properties of the object in question. From the age of one, children acquire increasingly complex cognitive and affective skills, leading to the conquest of language and, with it, the ability to play at a more imaginative and abstract level. Scholars point out that blind children remain engaged for a long time in play activities that include exploration of their bodies and solitary, nonfunctional, and aphinalistic manipulation of objects. In addition, pretend play appears with a significant delay compared to sighted peers, not appearing until 30–36 months (Besio et al., 2017).

Regarding symbolic ability in children with a congenital visual impairment, especially regarding the role of absent/impaired vision. Some authors believe that visual impairment is not a direct hindrance to the development of pretend play (Bishop et al., 2005; Lewis et al., 2000; Verver et al., 2020); others, instead, have observed limited pretend play behaviors (Celeste, 2006; Hughes et al., 1998; Tröster and Brambring, 1994). In support of the latter position, some studies suggest that children with blindness prefer solitary and more manipulative and exploratory play (Hughes et al., 1998; Tröster and Brambring, 1994). Hughes et al. (1998) identified a correlation between visual acuity and the type of play engaged in by blind children. Their findings indicated that blind children exhibited a preference for sensorimotor play. The authors hypothesized that resorting to visual information, even if partially, could promote the child’s involvement in pretend play and could increase their chances of interacting with peers. According to Lewis et al. (2000), the lack of access to social information can result in a disadvantage in pretend play skills, in relation to the inability to observe the behavior and interaction modes of others. However, this study highlighted how social skills and, therefore, the ability to relate to the surrounding world, may be related not only to visual impairment but also to other developmental variables and factors. In fact, when children who met the diagnostic criteria for autism were excluded from the analysis of the results, there were no significant differences between the pretend play expressed by blind children and that enacted by typically developing peers (Lewis et al., 2000). These findings are also in line with the results of Bishop et al. (2005). Furthermore, Verver et al. (2020) highlighted variability in pretend play capacity in relation to individual components such as social skills, linguistic skills, or the subject’s gender. They did not find a direct influence of visual impairment on pretend play.

From the analysis of the literature, a lack of agreement in the results achieved so far emerged, along with heterogeneity in the contexts and study settings used for the evaluation of pretend play and a limited use of standardized tools. These aspects, taken together, make it difficult to generalize the results. From these considerations, the need to deepen the research emerged.

The objective of the present study was thus to examine the development of pretend play skills in children between the ages of 3 and 9 years who had a peripheral visual impairment and were blind or had low vision, with the exclusion of central nervous system disorders or comorbidities, in comparison to typically developing children. Based on the results of the studies by Tröster and Brambring (1994), Hughes et al. (1998), and Celeste (2006), and in agreement with Pellegrini’s (2009) and Ferrell and Spungin’s (2011) reflections on pretend play and visual function, we hypothesized that—all things being equal—children who are blind or have a severe to moderate congenital visual impairment would have lower symbolic play skills and, therefore, lower scores on the Affect in Play Scale (APS) than typically developing children (Hypothesis 1 [H1]). Furthermore, if this difference is found, it is hypothesized that the play difficulty of visually impaired children would be proportional to the severity of the visual impairment (Hypothesis 2 [H2]).

Furthermore, since creativity is configured as a multidimensional construct analogous to symbolic play, as found in the studies of Moore and Russ (2008), Hoffmann and Russ (2012), Russ and Wallace (2013), and Russ (2014), a positive correlation between these constructs was hypothesized (Hypothesis 3 [H3]) for both groups. Finally, given that emotion regulation is a presumed and simultaneously preferred skill in the context of symbolic play and creativity (Berk et al., 2006; Moore and Russ, 2008; Russ and Wallace, 2013), we hypothesized (Hypothesis 4 [H4]) that higher emotion regulation scores would correspond to better symbolic play and creativity skills. Both groups in the study were analyzed.

## 2 Methods

### 2.1 Study design

The present study was based on a cross-sectional and observational (non-interventional) design, with multicentric convenience sampling. A non-randomized and controlled, between-subjects research design was adopted.

### 2.2 Materials

#### 2.2.1 Sociodemographics

A sociodemographic questionnaire was used to investigate the participant’s (i) assigned sex at birth; (ii) age; (iii) nationality; (iv) city of residence; (v) siblings; (vi) parent/guardians’ education level; and (vii) parent/guardians’ professions.

#### 2.2.2 Affect in play scale (APS)

To assess pretend play development, the APS was chosen. This instrument, validated and standardized, was created by psychologist and psychotherapist Sandra Russ (1993) and had been validated for the Italian population (Mazzeschi et al., 2016). The APS is a semi-structured test in which the experimenter provides instructions but allows the child to express themselves and organize the play. The play task involves the child playing with two fabric puppets (male/female) and three colored and shaped wooden blocks for 5 min. The play session should be video recorded to capture both narrative elements and non-verbal behaviors (e.g., facial expressions, prosodic narrative flow). The procedure commences with the verbatim transcription of the play task, which is then subjected to coding based on four cognitive categories and 11 affective categories. The cognitive categories include organization, elaboration, imagination, and comfort, rated on a Likert scale of 1–5 (1 = lowest score; 5 = highest score). The 11 affective categories identified by Sandra Russ include both negative and positive affectivity, enabling the calculation of theme frequency and variety concerning their specific valence (Mazzeschi et al., 2016). Regarding the APS (Russ, 1993), previous studies had reported high reliability among raters, and extensive validity and internal consistency (Mazzeschi et al., 2016; Russ, 2014).

#### 2.2.3 Storytelling task

As a measure of creativity, the consensual assessment technique devised by Hennessey and Amabile (1988) was adopted and applied to a storytelling task. Participants listened to the beginning of the audio story “Il gigante che aveva perso l’appetito- Fiabe per bambini [The giant who had lost his appetite: Fairy tales for children]” by Nieddu (2021) and were asked to continue the story, imagining what would happen next (the audio file is available on the Spotify platform).

The stories were scored by two independent evaluators. The evaluators did not use fixed criteria for scoring. For each story, they were asked to assign a score on a 1–5 Likert scale (1 = lowest score; 5 = highest score) for each of the dimensions identified by Hennessey and Amabile (1988): creativity, imagination, novelty, and likeability. Each story was subjectively rated in relation to the other stories produced. For the sake of clarity, the creativity dimension refers to how creative a story appeared to the evaluator (e.g., presence of dialogue, character names, plot developments); novelty refers to how rich a story appeared in novel elements; and imagination refers to the imaginative part of the story (e.g., fantastic scenarios). Finally, likeability refers to the overall likeability of the story told. In addition, a modification of the consensual rating technique was carried out, including a purely affective coding of the stories, using the 11 affective categories of the APS.

#### 2.2.4 Emotion regulation checklist (ERC)

To measure emotion regulation, the ERC-I the Italian version of the original ERC by Shields and Cicchetti (1997) was deemed appropriate. The ERC-I consists of 24 items designed to assess key emotional and regulatory processes on two factors: emotion regulation (e.g., *She/he is sensitive to others. She/he shows concern when others are upset or distressed*) and lability/negativity (e.g., *She/he shows great mood swings; it is difficult to predict the child’s emotional states, as she/he quickly goes from a positive mood to a negative one*). Emotion regulation is assessed by eight items that describe behaviors and emotional reactions that are consistent with contextual situations. Higher scores (maximum = 32) on this subscale indicate an adequate ability to manage and modulate one’s emotions in response to internal/external stimuli. The Lability/Negativity subscale includes 15 items that assess aspects of mood unpredictability and lack of regulation. In this case, higher scores (maximum = 60) indicate a state of greater dysregulation and therefore inappropriate emotional reactions to contexts and stimuli (Molina et al., 2014). Parents/tutors provide responses on a 4-point Likert scale, indicating the frequency of the behavior investigated by each item, ranging from 1 (almost always) to 4 (never).

### 2.3 Procedure and settings

The group of blind and visually impaired children (VIC) were recruited in Italy at the Developmental Neuro-ophthalmology Unit of the Scientific Institute for Research, Hospitalization, and Health Care Mondino Foundation in Pavia and at the Istituto David Chiossone Onlus in Genoa. The group of sighted children (SC) were recruited from the Municipal Playroom of Gubbio, Italy. All participants were volunteers and not compensated.

Participants were greeted individually in the administration room; time was spent familiarizing the children with the game materials and the administrators so that they would feel comfortable. Each center had an experiment room with the same following furnishings: a table and two chairs appropriate to the age of the children and a video camera positioned to film the entire play area.

The children completed two play tasks: the APS (Russ, 1993), followed by a storytelling task (Hennessey and Amabile, 1988). Their parents received an informative sheet about the research, including a module for informed consent declaration. Parents were also asked for authorization to use personal data, with a specific emphasis on using the data exclusively in aggregate form and following pseudonymization procedures. Additionally, parents completed a sociodemographic questionnaire and the ERC-I checklist.

### 2.4 Participants

The VIC included 31 preschool and school-aged children (3–9 years) with congenital blindness (9D90.6, 48.4%) or moderate (9D90.2, 25.8%) or severe (9D90.3, 25.8%) visual impairment according to the ICD-11. In the analyzed sample, 16 of the participants (51%) were male. One child was Romanian, one Egyptian, and one Moroccan; all the others were Italian (*n* = 28).

A total of 31 children with typical development were recruited to serve as a SC, matched to the VIC by sex and age. One child was Romanian; all the others were Italian. A detailed description of the experimental sample is available in the Supplementary Table S1.

### 2.5 Data analyses

Data analysis was performed using IBM® SPSS® Statistics 28.0.1 software. An inclusive criterion was adopted, retaining all data obtained. Null protocols were assigned a score of zero, considering non-play as a significant, measurable behavior comparable to other play behaviors. Descriptive statistics were initially calculated for each measure used. Furthermore, exploratory multivariate analyses (MANOVA) were conducted for both the VIC and SC. The independent variables included sex, socioeconomic status (SES), presence of siblings, and age, while all APS variables, storytelling, and the two ERC-I subscales were dependent variables. For the APS measure and storytelling task, the level of agreement was tested with Krippendorff’s coefficient (*α* ≥ 0.800, optimal reliability).

Paired samples *t*-tests were used to test symbolic play skills (H1) by comparing the SC and VIC participants. A multivariate analysis of variance (MANOVA) was used to test whether any play difficulties of the VIC children were directly proportional to the severity of visual impairment (H2).

Paired samples *t*-tests and partial correlation analyses were also used to test H3 and H4: *T*-tests were used to compare the two groups on creativity and emotion regulation. Partial correlations were used to test for the presence or absence of a correlation between creativity and symbolic play and emotion regulation within each group. Each analysis was examined while controlling for the variable “age.”

### 2.6 Ethical issues

The study received a favorable opinion from the University Committee on Bioethics of the University of Perugia and the Pavia Area Ethical Committee of the Institute of Scientific Research and Care “Policlinico San Matteo Foundation.” The study was conducted in accordance with the Helsinki Declaration, Good Clinical Practice Guidelines, and all current regulations regarding experimental studies. All parents of the participating children provided their informed consent to participate in the study. The first author (SF) collected the consents.

## 3 Results

The exploratory MANOVA yielded no significant evidence with regard to the independent variables of sex, socioeconomic status, and presence of siblings; however, a significant result was observed with respect to the variable “age,” both in the VIC group [Wilks’ Lambda = 0.000, *F*(108, 47) = 1.792, *p* = 0.013] and in the SC group [Wilks’ Lambda = 0.000, *F*(114, 41.869) = 1.722, *p* = 0.024]. Therefore, all subsequent analyses were conducted considering the age variable (see Supplementary Tables S2, S3).

### 3.1 H1: Pretend play between the blind and visually impaired children and sighted children

Comparing the scores obtained by the two groups (VIC, SC) on the APS measure by means of a paired-sample Student’s *t*-test, significant symbolic deficits were evident in the population of blind/visually impaired children analyzed here. In fact, the scores obtained by the VIC on the APS were significantly lower than those obtained by the SC. See Table 1.

**Table 1:** *T*-test results to compare pretend play (APS) between VIC and SC.

APS categories	VIC		SC		*t*	*p*	Cohen’s *d*
	*M*	*SD*	*M*	*SD*			
Organization	1.03	1.048	2.48	1.411	−5.229	<0.001	1.546
Elaboration	1.06	0.998	2.16	1.186	−4.697	<0.001	1.300
Imagination	1.16	1.128	2.35	1.279	−4.152	<0.001	1.600
Comfort	2.03	1.941	3.48	1.610	−3.594	<0.001	2.249
FREQ.TOT.AFF.	10.13	11.066	29.58	19.454	−4.878	<0.001	22.203
FREQ.POS.AFF.	7.19	8.072	18.16	14.459	−3.959	<0.001	15.424
FREQ.NEG.AFF.	2.94	4.312	11.10	11.155	−3.846	<0.001	11.816
VAR.TOT.AFF.	2.58	2.487	4.84	2.437	−3.791	<0.001	3.316
VAR.POS.AFF.	1.35	1.355	2.32	1.326	−2.997	0.003	1.798
VAR.NEG.AFF.	1.23	1.359	2.52	1.480	−3.927	<0.001	1.829

FREQ.TOT.AFF. = total frequency of affect; FREQ.POS.AFF. = frequency of positive affect; FREQ.NEG.AFF. = frequency of negative affect; VAR.TOT.AFF. = total variety of affect; VAR.POS.AFF. = variety of positive affect; VAR.NEG.AFF. = variety of negative affect.

### 3.2 H2: Pretend play and the severity of visual impairment

The MANOVA analysis used here did not provide statistically significant evidence of a directly proportional relationship between the severity of visual impairment and greater difficulty in playing, contrary to what was hypothesized (Supplementary Tables S2, S3). In other words, there was no pattern found where increased severity of visual impairment correlated with decreased play scores, both in cognitive and affective categories evaluated with the APS.

### 3.3 H3: Correlation between pretend play and creativity

First, the *t*-test analysis was carried out to compare the two groups on the storytelling task. The results showed significantly higher scores in the group of typically developing children (Table 2).

**Table 2:** *T*-test results to compare creativity (storytelling task) between VIC and SC.

Storytelling categories	VIC		SC		*t*	*p*	Cohen’s *d*
	*M*	*SD*	*M*	*SD*			
Creativity	1.13	1.204	2.35	1.582	−3.641	<0.001	1.875
Imagination	1.10	1.193	2.35	1.603	−3.917	<0.001	1.788
Novelty	1.00	1.211	2.35	1.561	−3.786	<0.001	1.992
Likeability	1.35	1.450	2.90	1.758	−3.886	<0.001	2.219
FREQ.TOT.AFF.	5.87	8.213	14.06	11.778	−3.136	0.002	14.545
FREQ.POS.AFF.	4.65	6.931	9.74	9.070	−2.445	0.010	11.608
FREQ.NEG.AFF.	1.23	1.875	4.32	4.650	−3.513	<0.001	4.908
VAR.TOT.AFF.	2.03	1.991	3.48	2.096	−3.087	0.002	2.618
VAR.POS.AFF.	1.39	1.334	2.06	1.209	−2.456	0.010	1.536
VAR.NEG.AFF.	1.23	1.359	1.42	1.285	−2.649	0.006	1.627

FREQ.TOT.AFF. = total frequency of affect; FREQ.POS.AFF. = frequency of positive affect; FREQ.NEG.AFF. = frequency of negative affect; VAR.TOT.AFF. = total variety of affect; VAR.POS.AFF. = variety of positive affect; VAR.NEG.AFF. = variety of negative affect.

Correlational analyses were then performed to investigate the relationship between pretend play and creativity, particularly in the form of narrative creativity, which is storytelling. Overall, positive correlations emerged between the APS results and per the storytelling task in both groups. Examining the results obtained in the VIC, we observed the cognitive APS variable “organization” significantly correlated with almost all the storytelling variables, particularly with the storytelling variables creativity [*r* (31) = 0.379, *p* < 0.05]; imagination [*r* (31) = 0.487, *p* < 0.01]; and likeability [*r* (31) = 0.462, *p* < 0.01]. Similarly, the APS variable “imagination” correlated with almost all the storytelling variables, including creativity [*r* (31) = 0.379, *p* < 0.05]; imagination [*r* (31) = 0.362, *p* < 0.05]; likeability [*r* (31) = 0.446, *p* < 0.05]; total frequency of affective themes [*r* (31) = 0.448, *p* < 0.05]; and total variety of affective themes [*r* (31) = 0.454, *p* < 0.05]. The variable “comfort” correlated significantly with the storytelling variable “likeability” [*r* (31) = 0.445, *p* < 0.05] and total variety of affective themes [*r* (31) = 0.370, *p* < 0.05]. The APS variable “total frequency of affective themes” correlated statistically with the storytelling variables creativity [*r* (31) = 0.451, *p* < 0.05], imagination [*r* (31) = 0.517, *p <* 0.01], novelty [*r* (31) = 0.444, *p* < 0.05], likeability [*r* (31) = 0.536, *p* < 0.05], total frequency of affective themes [*r* (31) = 0.484, *p* < 0.01], and total variety of affective themes [*r* (31) = 0.492, *p* < 0.01]. Finally, even the total variety of affective themes correlated with many of the storytelling variables, including creativity [*r* (31) = 0.385, *p* < 0.05]; imagination [*r* (31) = 0.411, *p* < 0.05]; likeability [*r* (31) = 0.482, *p* < 0.01]; and total frequency of affective themes [*r* (31) = 0.368, *p* < 0.05].

Regarding the SC, significant correlations were found between the APS variable “organization” and all the storytelling variables: creativity [*r* (31) = 0.675, *p* < 0.01]; imagination [*r* (31) = 0.659, *p* < 0.01]; novelty [*r* (31) = 0.609, *p* < 0.01]; likeability [*r* (31) = 0.628, *p* < 0.01]; total frequency of affective themes [*r* (31) = 0.544, *p* < 0.01]; and total variety of affective themes [*r* (31) = 0.482, *p* < 0.01]. We observed the same significant correlation for the APS variable “elaboration,” including for creativity [*r* (31) = 0.696, *p* < 0.01], imagination [*r* (31) = 0.657, *p* < 0.01], novelty [*r* (31) = 0.660, *p* < 0.01], likeability [*r* (31) = 0.642, *p* < 0.01], total frequency of affective themes [*r* (31) = 0.423, *p* < 0.05], and total variety of affective themes [*r* (31) = 0.523, *p* < 0.01]. The correlation was also very strong for the cognitive APS variable imagination; it was associated with the storytelling variables creativity [*r* (31) = 0.649, *p* < 0.01], imagination [*r* (31) = 0.613, *p* < 0.01], novelty [*r* (31) = 0.617, *p* < 0.01], likeability [*r* (31) = 0.631, *p* < 0.01], total frequency of affective themes [*r* (31) = 0.461, *p* < 0.05], and total variety of affective themes [*r* (31) = 0.568, *p* < 0.01]. The relationship between the APS variable “comfort” and the storytelling variables was also significant: creativity [*r* (31) = 0.603, *p* < 0.01], imagination [*r* (31) = 0.576, *p* < 0.01], novelty [*r* (31) = 0.622, *p* < 0.01], likeability [*r* (31) = 0.686, *p* < 0.01], total frequency of affective themes [*r* (31) = 0.479, *p* < 0.01], and total variety of affective themes [*r* (31) = 0.650, *p* < 0.01].

Significance was found for the APS variable “total frequency of affective themes” with creativity [*r* (31) = 0.473, *p* < 0.01], imagination [*r* (31) = 0.515, *p* < 0.01], novelty [*r* (31) = 0.582, *p* < 0.01], likeability [*r* (31) = 0.418, *p* < 0.01], total frequency of affective themes [*r* (31) = 0.475, *p* < 0.01], and total variety of affective themes [*r* (31) = 0.514, *p* < 0.01]. The same statistically significant relationship was observed between all the aforementioned storytelling variables and the affective APS variable “total variety of affective themes”; the storytelling variables were: creativity [*r* (31) = 0.507, *p* < 0.01], imagination [*r* (31) = 0.567, *p* < 0.01], novelty [*r* (31) = 0.612, *p* < 0.01], likeability [*r* (31) = 0.530, *p* < 0.01], total frequency of affective themes [*r* (31) = 0.483, *p* < 0.01], and total variety of affective themes [*r* (31) = 0.536, *p* < 0.01].

To identify which of the two groups exhibited stronger correlations, the results of the within-group correlations were transformed into Fisher’s *z*-scores. Then, a paired samples *t*-test was conducted to evaluate the mean differences between the internal correlations within each group (i.e., between-group analysis). The internal correlations within the SC were stronger than those of the VIC (Supplementary Table S4).

### 3.4 H4: Correlation between pretend play, creativity, and emotion regulation

A Student’s *t*-test was used to compare the scores of the two groups on the ERC-I. No statistically significant differences were found for emotion regulation, per the subscale Lability/Negativity, *t* = 0.825, *p* = 0.208, or the subscale Positive Regulation, *t* = −0.41, *p* = 0.484.

Partial Correlation was employed to investigate the relationship between pretend play, creativity, and emotion regulation within both groups. The results were negative for the subscale Lability/Negativity and positive for the subscale Positive Regulation, which followed the expected direction. For example, a negative score on the Lability/Negativity subscale indicated greater dysregulation and correlated with lower pretend play scores.

The Lability/Negativity subscale and APS scores were the main significant correlations that emerged from this analysis. Specifically, a statistically significant correlation was observed with these APS cognitive variables: organization [*r* (31) = −0.406, *p* < 0.05]; imagination [*r* (31) = −0.440, *p* < 0.05]; comfort [*r* (31) = −0.376, *p* < 0.05]; total frequency of affective themes [*r* (31) = −0.370, *p* < 0.05]; and total variety of affective themes [*r* (31) = −0.365, *p* < 0.05].

Concerning the variables related to storytelling, a significant correlation was observed between the Lability/Negativity subscale and total frequency of affective themes [*r* (31) = −0.370, *p* < 0.05] and total variety of affective themes [*r* (31) = −0.501, *p* < 0.01]. In the SC, scores followed the expected direction (negatively for the Lability/Negativity subscale; positively for the Positive Regulation subscale), but they did not correlate statistically significantly with the APS and storytelling variables examined. No between-group analyses were performed because there were no significant within-group correlations for the SC.

## 4 Discussion

This research explored the influence of visual impairments on symbolic skills by comparing differences in pretend play between children (aged 3–9 years) with blindness or moderate to severe visual impairments (VIC) and typically developing peers (SC). The typically developing children demonstrated superior pretend play skills compared to their blind and visually impaired peers. Furthermore, no differences were observed in the play performance of the VIC children based on the severity of visual impairment.

### 4.1 H1: Differences in pretend play between children with congenital blindness or visual impairments and peers with typical vision

The results of this study validated our research hypothesis by demonstrating a notable disparity between the two groups in terms of symbolic skills. The prevalence of zero scores in the VIC was noteworthy, suggesting that a significant portion of the studied cohort was unable to sustain a pretend play session. In addition, zero scores were observed also in the SC, although to a significantly lesser extent.

In general, the VIC children who were unable to engage in pretend play had difficulty establishing a relationship with the experimenter and tended to withdraw from the context and the task of play. Two contrasting patterns of behavior were observed in these children: on the one hand, traits indicative of inhibition and introversion; on the other hand, traits suggestive of inattention and hyperactivity.

Also, the VIC children scored significantly lower than the SC children. This demonstrated the greater difficulty in pretend play for children with a congenital visual impairment and underscores the importance of vision in the development of symbolic skills, in line with Ferrell and Spungin’s (2011) reflections. This was evidenced by their inability to create mental connections that allowed them to apply previously learned concepts to new situations. A deficiency in these cognitive abilities results in notable deficiencies in imaginative capacity.

In our study, both groups engaged in storytelling related to everyday life. However, the children with typical development exhibited a greater degree of creativity in their storytelling. Their narratives were characterized by the inclusion of fragments of previously heard stories, reflecting an internalization and integration of content. This contributed to the cohesion of the narrative fabric. Furthermore, they demonstrated a greater capacity for structuring, as evidenced by the introduction of multiple characters (human and non-human), a diversification of contexts and scenarios, and the use of distinct voices for each character.

An essential aspect regarding the VIC was related to the more functional use of play materials, particularly wooden blocks, in accordance with the arguments put forth by Lewis et al. (2000). These materials are rarely subjected to mental transformations—manipulations typical of symbolism—and thus are seldom utilized by visually impaired children to represent something else.

The attribution of alternative meanings is facilitated by imitative processes and the possibility of finding elements of inspiration in others, which necessarily originate from vision. During our administrations, the difficulties of children with visual impairments to attribute symbolic meanings were evident from the outset, with the first contact with the play materials. They would have preferred to use sound-tactile materials that allowed them to experience the object through multiple sensory channels, findings which were also noted by Tröster and Brambring (1994) and Hughes et al. (1998).

A shift in focus towards an emotional perspective revealed a wider and more varied range of emotional themes in the APS stories produced by the SC children. They appeared to be more proficient in recreating feelings and emotions experienced within the context of play, utilizing this as a tool to rework their reality. In contrast, the visually impaired children exhibited a markedly lower quantity and diversity of emotional themes, a finding that was further substantiated by our quantitative analyses. This could indicate that the different modes of storing life experiences and situations result in memories being less vivid and poignant. Consequently, it becomes more challenging to recreate emotionally connoted themes in play.

The relevance of reflections on play behaviors is further underscored when considering that the VIC children were enrolled in a rehabilitation program in which play and the acquisition of relational skills are central. Despite the clinical attention that has been directed towards these areas of development, vulnerabilities still emerge. This suggests the need for early intervention to accompany the child in their growth journey, starting from the first months of life. The newborn infant is indeed a dynamic and open system that, through continuous exchanges with others, increases in complexity, giving rise to meanings about the self, world, and self-world (Tronick and Beeghly, 2011).

### 4.2 H2: The difficulty of play and visual impairment severity

The hypothesis that there would be a significant relationship between the severity of visual impairment and pretend play abilities was not borne out by the findings. In particular, there was no evidence that children with congenital blindness exhibited inferior play abilities than children with a severe or moderate visual impairment. The absence of significant differences in play among participants with blindness or a severe or moderate visual impairment can be partly explained by the minimal residual visual functionality that some children exhibit in near distance vision. This functionality allows blind children to explore the peri-personal space and behave in play similarly to children with visual impairment. However, this consideration is incomplete, given the presence of children with no residual vision. Therefore, the results obtained require further elucidation.

First, perhaps the primary impact of visual impairment, whether severe or moderate, is not determined by the severity of the impairment itself, but rather by its presence or absence. If we consider the fact, as discussed in the Introduction, that in children without disabilities, the visual system takes in approximately 80% of the sensory information (Ferrell and Spungin, 2011), it is reasonable to conclude that an effect on perceptual and symbolic abilities is already apparent with low vision and not only with total blindness. Therefore, the depth of impairment presented by severely and moderately visually impaired children is such that it equates them behaviorally and cognitively to blind children. This indicates that to highlight significant differences in play behavior, the critical level should be set above the residual visual acuity corresponding to 2/10, thus including subjects with less severe visual impairment (e.g., mild visual impairment).

Second, it is crucial to acknowledge the role of subjectivity and individual variability in determining the developmental pathways of each person, as also emphasized by Ferrell and Smyth (2017). This may have been overlooked due to the limited number of participants included in the VIC.

### 4.3 H3: The relationship between pretend play and creativity

Our findings revealed a significant correlation between pretend play and creativity. This indicates a degree of agreement between the results from the APS measure and the storytelling task, both in the blind and visually impaired children and sighted children. Nevertheless, it is crucial to direct attention toward the discrepancies in performance between the two groups in the storytelling task. Similar to the results obtained with the APS, the SC reported significantly higher outcomes. Moreover, a considerable number of children who did not proceed to perform the play task did not engage in the storytelling task either. These findings indicate a similarity in the underlying patterns of the two constructs, as previously suggested in studies by Moore and Russ (2008), Hoffmann and Russ (2012), Russ and Wallace (2013), and Russ (2014). This allows for inferring the influence of congenital visual impairment on narrative creativity. In the APS and storytelling tasks, the tactile and auditory channels, respectively, are the preferred channels of input. However, the poor results obtained in both tasks indicate that the mode of presentation of the stimulus is irrelevant for blind or visually impaired children. This suggests that the acquisition of knowledge about the world, which may have been hindered by congenital visual impairment, is necessary for symbolic and creative engagement.

It is evident that the children in the VIC created less complex and imaginative stories. This reduced ability to invent new and varied stories can be attributed to the limited ability of these children to explore the surrounding physical space. It is well-established that spatial skills are negatively influenced by sensory difficulties, particularly visual ones, which hinder the Gestaltic and immediate perception of the world around them (Morelli et al., 2020). This will also have an impact on the child’s cognitive development, as well as their capacity to imagine potential scenarios (Ferrell and Spungin, 2011).

Moreover, the narratives created by the blind or visually impaired children were distinguished by a singular, predominantly external narrative voice that precluded dialogue between characters and alterations in voice. This may indicate a lack of or poor sense of identification, which could be defined as a deficient ability to empathize with the characters of the story, in this case the giant and the fairy. The opening of the proposed fairy tale is replete with expressions that reflect the emotional state of the protagonists, thereby prompting the subject to devise potential solutions through the application of theory of mind-related competencies. Previous studies had reported a difficulty of visually impaired children in recognizing others’ emotions (Dyck et al., 2004). This difficulty is hypothesized to be due to the inability to perceive highly salient visual elements. Non-verbal elements such as postures, gestures, and facial expressions are particularly challenging to comprehend in the absence of sight. This appears to result in impairments in mind reading and difficulties in using visual imitation in playful and creative contexts (Grumi et al., 2021).

### 4.4 H4: Emotion regulation

The results from the ERC-I indicated no notable discrepancy between the blind and visually impaired children and sighted children, suggesting that the presence of a visual impairment does not inherently correlate with heightened emotion dysregulation. Nevertheless, a significant inverse correlation was observed between the constructs of play and creativity and emotion regulation within the VIC. Specifically, it was found that children with lower play scores also had higher scores on the Lability/Negativity subscale, indicating greater emotion dysregulation. This finding is relevant because, upon intra-group analysis, it was found that children with greater emotion dysregulation exhibited lower play scores. This result is consistent with the theoretical framework proposed by Russ (1993, 2014), which posits that play-creative skills are contingent upon emotion regulation abilities, and vice versa. In light of the suboptimal performance of the VIC on the APS and storytelling tasks, it is proposed that a rehabilitative intervention focused on emotion regulation could enhance pretend play and creativity.

### 4.5 Study limitations

First, it is necessary to emphasize the heterogeneity of the settings in which the experiments were conducted. Although both groups were in familiar environments, it would be advisable to standardize the setting to ensure consistency. Also, as suggested by one reviewer, it would be useful for future studies to examine participants’ familiarity with pretend play acquired during children’s development, for example, during preschool activities or story reading by family members. Furthermore, in order to enhance the reliability of the results, it would be advisable to expand the sample size. With regard to methodological limitations, the use of a parent-report instrument may have introduced bias, potentially leading to either overestimation or underestimation of the child’s emotion regulation abilities by the parent or guardian. Consequently, the utility of a multimethod and multiple-informant approach is recommended.

### 4.6 Implications for further research

A prospective line of inquiry might involve the examination of pretend play from a developmental perspective, utilizing a longitudinal study design. Second, a future study could extend research on creativity in children with congenital visual impairments by incorporating a test of divergent thinking into the storytelling task. A further crucial aspect to be addressed is the potential impact of individual characteristics on play behavior. In this study, children with neurodevelopmental disorders, such as autism spectrum disorder or attention deficit hyperactivity disorder, were excluded. However, the study design did not allow for the control of behavioral traits that—although not constituting a separate diagnosis—represent specific modes of individual functioning. Moreover, it would be beneficial to ascertain potential correlations between the etiology of visual disability and specific behavioral traits, with the aim of elucidating the manner in which visual impairment affects brain architecture and functionality. This would facilitate the design and implementation of an individualized intervention plan that is carefully tailored to the emotional and cognitive functioning of the individual.

## 5 Conclusion

In this study we extensively examined the differences in pretend play between typically developing children and children with congenital visual impairments, using a standardized and validated tool that had never before been used for this specific population. The findings illuminated crucial elements that elucidate the influence of visual impairment on pretend play abilities. Furthermore, they have established a foundation for prospective investigations into the correlates of creativity and emotion regulation in individuals diagnosed with these conditions.

As hypothesized, the group of children with a visual impairment exhibited significant deficits in pretend play. Nevertheless, no correlations were observed between the degree of impairment and play competence, as evidenced by within-group analyses. The children with a visual impairment exhibited difficulties not only in pretend play but also in creative competence, particularly in narrative aspects. This supports the hypothesis of an interconnection between the two constructs and a similarity in underlying patterns. However, the results indicated that visual impairment did not appear to influence emotion regulation, as the VIC did not score lower than the SC. Conversely, the data indicated a negative correlation between emotion dysregulation and performance in play and storytelling in VIC, emphasizing that greater dysregulation is associated with poorer play abilities. This allows for the inference that play and emotion regulation are interconnected and bidirectional, as action on one can have significant effects on the other.

Focusing a re-habilitation intervention on enhancing play skills, particularly pretend play skills, means promoting the development of the individual as a whole, supporting both cognitive and emotional dimensions. The play space becomes a training ground for flexible thinking, problem-solving skills, and the acquisition of metacognitive abilities, as well as a safe environment for exploring the self-other relationship. Indeed, it allows for gaining new perspectives, engaging with one’s own and others’ emotional dimensions, and thus strengthening self-regulatory abilities and the ability to interpret others’ mental and emotional states.

Considering the limited attention in the literature on the topic of play and creativity in the context of visual impairment, our study emphasizes these aspects and their importance in therapeutic contexts. Play emerges as a versatile tool with dual utility. On the one hand, it allows for the observation of the internal emotional-cognitive world of the child. On the other hand, it serves as a potent clinical-rehabilitative tool aimed at enhancing cross-cutting skills closely linked to the adaptive functioning of each individual. These considerations provide a foundation for future research and studies aimed at advancing understanding and proximity to the topic of visual impairment.

## Data Availability

The raw data supporting the conclusions of this article will be made available by the authors, without undue reservation.

## References

[ref1] Archinto F. (2001). Symbolic play and narrative skills: similarities and differences. J. Educ. Technol. 9, 42–47. doi: 10.17471/2499-4324/559

[ref2] Berk L. E. Mann T. D. Ogan A. T. (2006). “Make-believe play: wellspring for development of self-regulation” in Play = learning: How play motivates and enhances children's cognitive and social-emotional growth. eds. Singer D. G. Golinkoff R. M. Hirsh-Pasek K. (Oxford, UK: Oxford University Press), 74–100.

[ref3] Besio S. Bulgarelli D. Stancheva-Popkostadinova V. (2017). Play development in children with disabilities: Warsaw, PL. De Gruyter Open.

[ref4] Bishop M. Hobson R. P. Lee A. (2005). Symbolic play in congenitally blind children. Dev. Psychopathol. 17, 447–465. doi: 10.1017/S0954579405050212, PMID: 16761553

[ref5] Celeste M. (2006). Play behaviors and social interactions of a child who is blind: in theory and practice. J. Visual Impairment Blindness 100, 75–90. doi: 10.1177/0145482X0610000203

[ref6] Chennaz L. Valente D. Baltenneck N. Baudouin J.-Y. Gentaz E. (2022). Emotion regulation in blind and visually impaired children aged 3 to 12 years assessed by a parental questionnaire. Acta Psychol. 225:103553. doi: 10.1016/j.actpsy.2022.103553, PMID: 35279432

[ref7] Dale N. Sakkalou E. O'Reilly M. Springall C. De Haan M. Salt A. (2017). Functional vision and cognition in infants with congenital disorders of the peripheral visual system. Dev. Med. Child Neurol. 59, 725–731. doi: 10.1111/dmcn.13429, PMID: 28439876

[ref8] Dyck M. J. Farrugia C. Shochet I. M. Holmes-Brown M. (2004). Emotion recognition/understanding ability in hearing or vision-impaired children: do sounds, sights, or words make the difference? J. Child Psychol. Psychiatry 45, 789–800. doi: 10.1111/j.1469-7610.2004.00272.x, PMID: 15056310

[ref9] Fazzi E. Lanners J. Ferrari-Ginevra O. Achille C. Luparia A. Signorini S.. (2002). Gross motor development and reach on sound as critical tools for the development of the blind child. Brain Dev 24, 269–275. doi: 10.1016/S0387-7604(02)00021-9, PMID: 12142062

[ref10] Fein G. G. (1987). “Pretend play: creativity and consciousness” in Curiosity, imagination, and play: On the development of spontaneous cognitive motivational processes. eds. Görlitz D. Wohlwill J. F. (Hillsdale, US: Lawrence Erlbaum Associates), 281–304.

[ref11] Ferrell K. A. Smyth C. A. (2017). “Growth and development of young children” in Foundations of education, third edition, volume i: History and theory of teaching children and youths with visual impairments. eds. Holbrook M. C. McCarthy T. Kamei-Hannan C.. 3rd ed (New York, NY: AFB Press), 113–145.

[ref12] Ferrell K. A. Spungin S. J. (2011). Reach out and teach: helping your child who is visually impaired learn and grow, New York, NY: AFB Press.

[ref13] Freud A. (1965). The writings of anna freud, vol. 6: New York, NY. International Universities Press.

[ref14] Golomb C. Galasso L. (1995). Make believe and reality: explorations of the imaginary realm. Dev. Psychol. 31, 800–810. doi: 10.1037/0012-1649.31.5.800

[ref15] Grumi S. Cappagli G. Aprile G. Mascherpa E. Gori M. Provenzi L.. (2021). Togetherness, beyond the eyes: a systematic review on the interaction between visually impaired children and their parents. Infant Behav. Dev. 64:101590. doi: 10.1016/j.infbeh.2021.101590, PMID: 34062369

[ref16] Hennessey B. A. Amabile T. M. (1988). Story-telling: a method for assessing children’s creativity. J. Creat. Behav. 22, 235–246. doi: 10.1002/j.2162-6057.1988.tb00502.x

[ref17] Hoffmann J. Russ S. (2012). Pretend play, creativity, and emotion regulation in children. Psychol. Aesthet. Creat. Arts 6, 175–184. doi: 10.1037/a0026299

[ref18] Hoffmann J. D. Russ S. W. (2016). Fostering pretend play skills and creativity in elementary school girls: a group play intervention. Psychol. Aesthet. Creat. Arts 10, 114–125. doi: 10.1037/aca0000039

[ref19] Hughes M. Dote-Kwan J. Dolendo J. (1998). A close look at the cognitive play of preschoolers with visual impairments in the home. Except. Child. 64, 451–462. doi: 10.1177/001440299806400402

[ref20] Lewis V. Norgate S. Collis G. Reynolds R. (2000). The consequences of visual impairment for children's symbolic and functional play. Br. J. Dev. Psychol. 18, 449–464. doi: 10.1348/026151000165797

[ref21] Mazzeschi C. Salcuni S. Di Riso D. Chessa D. Delvecchio E. Lis A.. (2016). E tu giochi. La valutazione del gioco simbolico in età evolutiva: L’affect in play scale [and you play. The assessment of symbolic play in developmental age: the affect in play scale.]: Milan, IT. Franco Angeli.

[ref22] Molina P. Sala M. N. Zappulla C. Bonfigliuoli C. Cavioni V. Zanetti M. A.. (2014). The emotion regulation checklist – Italian translation. Validation of parent and teacher versions. Eur. J. Dev. Psychol. 11, 624–634. doi: 10.1080/17405629.2014.898581

[ref23] Moore M. Russ S. W. (2008). Follow-up of a pretend play intervention: effects on play, creativity, and emotional processes in children. Creat. Res. J. 20, 427–436. doi: 10.1080/10400410802391892

[ref24] Morelli F. Aprile G. Cappagli G. Luparia A. Decortes F. Gori M.. (2020). A multidimensional, multisensory and comprehensive rehabilitation intervention to improve spatial functioning in the visually impaired child: a community case study. Front. Neurosci. 14:768. doi: 10.3389/fnins.2020.00768, PMID: 32792904 PMC7393219

[ref25] Nieddu R. (2021). Il Gigante che aveva perso l’appetito: Fiabe per bambini [the giant who had lost his appetite: fairy tales for children]. Available online at: https://open.spotify.com/episode/3LwtR7vEdNHve1zS1tzdQA (Accessed June 12, 2024).

[ref26] Pellegrini A. D. (2009). The role of play in human development: New York, NY. Oxford University Press.

[ref27] Piaget J. (1964). Six études de psychologie [Six psychological studies] Paris, FR: Folio Essays.

[ref28] Russ S. W. (1993). Affect and creativity: The role of affect and play in the creative process: Hillsdale, NJ. Lawrence Erlbaum Associates.

[ref29] Russ S. W. (2014). Pretend play in childhood: Foundation of adult creativity: Washington, DC: American Psychological Association.

[ref30] Russ S. W. Wallace C. E. (2013). Pretend play and creative processes. Am. J. Play 6, 136–148.

[ref31] Shields A. Cicchetti D. (1997). Emotion regulation among school-age children: the development and validation of a new criterion q-sort scale. Dev. Psychol. 33, 906–916. doi: 10.1037/0012-1649.33.6.906, PMID: 9383613

[ref32] Shields A. Cicchetti D. (1998). Reactive aggression among maltreated children: the contributions of attention and emotion dysregulation. J. Clin. Child Psychol. 27, 381–395. doi: 10.1207/s15374424jccp2704_2, PMID: 9866075

[ref33] Stagnitti K. (2004). Understanding play: the implications for play assessment. Aust. Occup. Ther. J. 51, 3–12. doi: 10.1046/j.1440-1630.2003.00387.x

[ref34] Tronick E. Beeghly M. (2011). Infants’ meaning-making and the development of mental health problems. Am. Psychol. 66, 107–119. doi: 10.1037/a0021631, PMID: 21142336 PMC3135310

[ref35] Tröster H. Brambring M. (1994). The play behavior and play materials of blind and sighted infants and preschoolers. J. Visual Impairment Blindness 88, 421–432. doi: 10.1177/0145482X19948805008

[ref36] Verver S. H. Vervloed M. P. J. Steenbergen B. (2020). Characteristics of peer play in children with visual impairments. Res. Dev. Disabil. 105:103714. doi: 10.1016/j.ridd.2020.103714 32623248

[ref37] Warren D. H. (1994). Blindness and children: An individual differences approach: New York, NY. Cambridge University Press.

[ref38] World Health Organization. (2019). World report on vision. Available online at: www.who.int/publications-detail/world-report-on-vision (Accessed June 12, 2024).

[ref39] World Health Organization (2019/2021). International classification of diseases, eleventh revision (ICD-11). 11th Edn: Geneva, CH. WHO.

