# Supplementary material for: Pretend play in children with a congenital visual impairment
Source: Front Psychol. 2025 Apr 30;16:1535086. doi: 10.3389/fpsyg.2025.1535086 (PMC12075109; doi:10.3389/fpsyg.2025.1535086)
Supplement: Supplementary file 1 [file Supplementary_file_1.docx]

**Supplementary material**

to

**Pretend play in children with congenital visual impairment**

by

SF, AB, CB, ED, AL, FM, IS, EC, LS, SS^^[[1]](#footnote-1)^^

## **Table S1**

*In the present table, the diagnostic specifics of the VIC are provided. The first column indicates the alphanumeric code identifying each participant, while the second column specifies their age, followed by gender in the third column. The fourth column references the specific diagnosis assigned to the individual, followed by their visual residue.*

| VIC | Age | Sex | Diagnosis | BCVA (logMAR) |
| --- | --- | --- | --- | --- |
| AB01-7-1 | 7 | M | Ocular Albinism | 0.80 |
| CB02-6-2 | 6 | F | Optic Nerve Hypoplasia | Blindness |
| AB03-7-2 | 7 | F | Retinopathy of prematurity | 0.80 |
| CB04-8-1 | 8 | M | Bilateral congenital retinoblastoma, monocular vision | 1.30 |
| AB05-5-2 | 5 | F | Ocular coloboma | 0.80 |
| CB06-5-1 | 5 | M | Inherited retinal dystrophy | Blindness |
| AB07-6-1 | 6 | M | Congenital nystagmus | 1.00 |
| AB08-8-1 | 8 | M | Inherited retinal dystrophy | Blindness |
| CB09-9-2 | 9 | F | Inherited retinal dystrophy | Blindness |
| AB10-7-1 | 7 | M | Inherited retinal dystrophy | Blindness |
| CB11-3-2 | 3 | F | Inherited retinal dystrophy | 1.00 |
| AB12-2-1 | 3 | M | Bilateral Iridal and Choroidal Retinal Coloboma | 1.00 |
| CB13-7-1 | 7 | M | Bilateral congenital cataract | 0.80 |
| AB14-7-1 | 7 | M | Inherited retinal dystrophy | Blindness |
| CB15-5-1 | 5 | M | Inherited retinal dystrophy | Blindness |
| AB16-6-2 | 6 | F | Inherited retinal dystrophy | Blindness |
| CB17-4-2 | 4 | F | Anophthalmia | Blindness |
| CB19-7-2 | 7 | F | Retinopathy of prematurity | Blindness |
| AB20-7-2 | 7 | F | Inherited retinal dystrophy | 1.00 |
| CB21-5-2 | 5 | F | Ocular Albinism | 1.00 |
| AB22-6-2 | 6 | F | Ocular Albinism | 0.70 |
| CB23-6-1 | 6 | M | Congenital Glaucoma | Blindness |
| AB24-5-1 | 5 | M | Inherited retinal dystrophy | Blindness |
| CB25-4-1 | 4 | M | Congenital Cataract | 0.80 |
| AB26-5-2 | 5 | F | Retinopathy of prematurity | 0.80 |
| CB27-7-1 | 7 | M | Hypoplasia of the Optic Nerves, Chiasm, and Optic Pathways | Blindness |
| AB28-7-1 | 7 | M | Chorioretinal and Iridal Coloboma | 1.00 |
| CB29-7-1 | 7 | M | Congenital Glaucoma | Blindness |
| AB30-8-2 | 8 | F | Inherited retinal dystrophy | 1.30 |
| CB31-3-2 | 3 | F | Inherited retinal dystrophy | Blindness |
| AB32-3-2 | 3 | F | Macular dystrophy | 0.70 |

Note: Visual acuity was categorised as mild (0.70-0.50 logMAR), moderate (0.70-1 logMAR), severe (1-1.30 logMAR) low vision or blindness (>1.30 logMAR).

## **Table S2**

*Results of MANOVA (Multivariate Analysis of Variance) for APS Cognitive variables in relation to visual category, controlling the variable “age”.*

| **Effect** | | **Value** | **F** | **Hypothesis df** | **Error df** | **Sig.** |
| --- | --- | --- | --- | --- | --- | --- |
| VIC_VISUAL_CATEGORY * VIC_AGE | Pillai's Trace | 0.447 | 1.653 | 8.000 | 46.000 | 0.136 |
|  | Wilks' Lambda | 0.600 | 1.602^b^ | 8.000 | 44.000 | 0.152 |
|  | Hotelling's Trace | 0.590 | 1.549 | 8.000 | 42.000 | 0.170 |
|  | Roy's Largest Root | 0.394 | 2.265^c^ | 4.000 | 23.000 | 0.093 |

*Note*. b. Exact statistic; c. The statistic is an upper bound on F that yields a lower bound on the significance level.

| **Source** | | **Type III Sum of Squares** | **df** | **Mean Square** | **F** | | **Sig.** |
| --- | --- | --- | --- | --- | --- | --- | --- |
| VIC_VISUAL_  CATEGORY* VIC_AGE | VIC_APS_ORG | 0.645 | 2 | 0.322 | | 0.446 | 0.645 |
|  | VIC_APS_ELAB | 1.663 | 2 | 0.832 | | 1.594 | 0.223 |
|  | VIC_APS_IMAG | 1.854 | 2 | 0.927 | | 1.192 | 0.320 |
|  | VIC_APS_COMFORT | 10.246 | 2 | 5.123 | | 2.115 | 0.142 |
|  |  |  |  |  | |  |  |

*Note*: VIC_APS_ORG = APS Organization in blind and visually impaired children group; VIC_APS_ELAB = APS Elaboration in blind and visually impaired children group; VIC_APS_IMAG = APS Imagination in blind and visually impaired children group; VIC_APS_COMFORT = APS Comfort in blind and visually impaired children group; b. Exact statistic; c. The statistic is an upper bound on F that yields a lower bound on the significance level.

## **Table S3**

*Results of MANOVA (Multivariate Analysis of Variance) for APS Affective variables in relation to*

*visual category, controlling the variable “age”.*

| **Effect** | | **Value** | **F** | **Hypothesis df** | **Error df** | **Sig.** |
| --- | --- | --- | --- | --- | --- | --- |
| VIC_VISUAL_CATEGORY* VIC_AGE | Pillai's Trace | 0.290 | 0.974 | 8.000 | 46.000 | 0.468 |
|  | Wilks' Lambda | 0.723 | .969^b^ | 8.000 | 44.000 | 0.472 |
|  | Hotelling's Trace | 0.366 | 0.961 | 8.000 | 42.000 | 0.479 |
|  | Roy's Largest Root | 0.311 | 1.787^c^ | 4.000 | 23.000 | 0.166 |

*Note*. b. Exact statistic; c. The statistic is an upper bound on F that yields a lower bound on the significance level.

| **Source** | | **Type III Sum of Squares** | **df** | **Mean Square** | **F** | | **Sig.** |
| --- | --- | --- | --- | --- | --- | --- | --- |
| VIC_VISUAL_  CATEGORY  * VIC_AGE | VIC_APS_FREQ_TOT_AFFECTS | 202.671 | 2 | 101.336 | | 1.130 | 0.339 |
|  | VIC_APS_FREQ_POS_AFFECTS | 81.698 | 2 | 40.849 | | 0.832 | 0.447 |
|  | VIC_APS_ FREQ_NEG_AFFECTS | 41.720 | 2 | 20.860 | | 1.261 | 0.301 |
|  | VIC_APS_VAR_TOT_  AFFECTS | 14.349 | 2 | 7.175 | | 1.712 | 0.201 |
|  | VIC_APS_VAR_POS_  AFFECTS | 4.419 | 2 | 2.210 | | 1.649 | 0.212 |
|  | VIC_APS_VAR_NEG_  AFFECTS | 2.854 | 2 | 1.427 | | 0.983 | 0.388 |

*Note.* VIC_APS_FREQ_TOT_AFFECTS = group of blind and visually impaired children APS total frequency of affects; VIC_APS_FREQ_POS_AFFECTS = group of blind and visually impaired children APS frequency of positive affects; VIC_APS_FREQ_NEG_AFFECTS = group of blind and visually impaired children APS frequency of negative affects; VIC_APS_VAR_TOT_AFFECTS = group of blind and visually impaired children APS total variety of affects; VIC_APS_VAR_POS_AFFECTS = group of blind and visually impaired children APS variety of positive affects; VIC_APS_VAR_NEG_AFFECTS = group of blind and visually impaired children APS variety of negative affects; b. Exact statistic; c. The statistic is an upper bound on F that yields a lower bound on the significance level.

## **Table S4**

*Paired samples T-test used to assess the mean differences between the internal correlations within each group (VIC vs SC).*

| Categories | *VIC* | | *SC* | | *t* | *p* | Cohen’s *d* |
| --- | --- | --- | --- | --- | --- | --- | --- |
|  | *M* | *SD* | *M* | *SD* |  |  |  |
| APS_ORG_all_Story | .475 | .0701 | .699 | .112 | -3.530 | .008 | .15515 |
| APS_ELAB_all_Story | .369 | .0687 | .705 | .156 | -4.715 | .003 | .17486 |
| APS_IMAG_all_Story | .424 | .070 | .682 | .099 | -4.267 | .004 | .14799 |
| APS_COMFORT_all_Story | .347 | .062 | .703 | .109 | -7.950 | <.001 | .10980 |
| APS_FREQ_all_Story | .533 | .047 | .546 | .074 | -.292 | .391 | .10928 |
| APS_VAR_all_Story | .423 | .065 | .605 | .082 | -4.447 | .003 | .10025 |

*Note*: APS_ORG_all_Story = APS Organization and all storytelling variables; APS_ELAB_all_Story = APS Elaboration and all storytelling variables; APS_IMAG_all_Story = APS Imagination and all storytelling variables; APS_COMFORT_all_Story = APS Comfort and all storytelling variables; APS_FREQ_all_Story = APS Total frequency of affect and all storytelling variables; APS_VAR_all_Story = Total variety of affect and all storytelling variables.

1. **Contact Information**: Sabrina Signorini, via Mondino, 2, 27100 Pavia; sabrina.signorini@mondino.it [↑](#footnote-ref-1)
